# Pregnant women and health workers’ perspectives on perinatal mental health and intimate partner violence in rural Ethiopia: a qualitative interview study

**DOI:** 10.1186/s12884-023-05352-8

**Published:** 2023-01-28

**Authors:** Roxanne C. Keynejad, Tesera Bitew, Adiyam Mulushoa, Louise M. Howard, Charlotte Hanlon

**Affiliations:** 1 Section of Women’s Mental Health, Health Service and Population Research Department, PO31 David Goldberg Building, Institute of Psychiatry, Psychology & Neuroscience, King’s College London, Denmark Hill, London, SE5 8AF UK; 2 Department of Psychiatry, School of Medicine, College of Health Sciences, Addis Ababa University, Addis Ababa, Ethiopia; 3 Centre for Innovative Drug Development and Therapeutic Trials for Africa (CDT-Africa), College of Health Sciences, Addis Ababa University, Addis Ababa, Ethiopia; 4 Centre for Global Mental Health, Health Service and Population Research Department, PO31 David Goldberg Building, Institute of Psychiatry, Psychology & Neuroscience, King’s College London, Denmark Hill, London, SE5 8AF UK

**Keywords:** Pregnancy, Perinatal mental health, Perinatal depression, Antenatal care, Intimate partner violence, Domestic violence, Ethiopia, Qualitative, Interviews

## Abstract

**Background:**

Mental health conditions are common during the perinatal period and associated with maternal, foetal, and neonatal morbidity and mortality. There is an established bidirectional relationship between mental health conditions and intimate partner violence (IPV), including during and after pregnancy. Mean lifetime prevalence of physical, sexual or emotional IPV exposure among women in rural Ethiopia is estimated to be 61% and may be even higher during the perinatal period. We aimed to explore the perspectives of women and antenatal care (ANC) health workers on the relationship between all types of IPV and perinatal mental health, to inform the adaptation of a psychological intervention for pregnant women experiencing IPV in rural Ethiopia.

**Methods:**

We conducted in-depth qualitative interviews with 16 pregnant women and 12 health workers in the Gurage zone of the Southern Nations, Nationalities and People’s Region of Ethiopia, between December 2018 and December 2019. We conducted thematic analysis of English-translated transcripts of audio-recorded Amharic-language interviews.

**Results:**

Participants contextualised IPV as the primary form of abusive treatment women experienced, connected by multiple pathways to emotional and bodily distress. Patriarchal norms explained how the actions of neighbours, family, community leaders, law enforcement, and government agents in response to IPV often reinforced women’s experiences of abuse. This created a sense of powerlessness, exacerbated by the tension between high cultural expectations of reciprocal generosity and severe deprivation. Women and health workers advocated a psychological intervention to address women’s powerlessness over the range of difficulties they faced in their daily lives.

**Conclusions:**

Women and health workers in rural Ethiopia perceive multiple, interconnected pathways between IPV and perinatal emotional difficulties. Contrary to expectations of sensitivity, women and health workers were comfortable discussing the impact of IPV on perinatal mental health, and supported the need for brief mental health interventions integrated into ANC.

**Supplementary Information:**

The online version contains supplementary material available at 10.1186/s12884-023-05352-8.

## Background

The World Health Organization (WHO) recognises violence against women as a major public health concern, mostly comprising intimate partner violence (IPV) [1]. IPV is behaviour by an intimate partner or ex-partner, which causes physical, sexual, or psychological harm [1]. A WHO multi-country study [2] of 24,000 women across 10 low, middle, and high-income countries found high prevalence estimates of physical (13–61%), sexual (6–59%), and psychological (20–75%) IPV.

IPV during pregnancy is associated with adverse health outcomes, including obstetric and neonatal risks, mental health conditions, suicide, and femicide [3, 4]. Understanding stakeholders’ perspectives on the relationship between IPV and perinatal mental health is an important first step to developing and adapting interventions integrated into antenatal care (ANC).

A systematic review of 10 studies found a mean lifetime prevalence of any IPV in Ethiopia among women aged 15–49 years of 61% [5], constituting 48% physical IPV, 40% sexual IPV, and 52% emotional IPV. A meta-analysis of eight studies (*n* = 2691 women) found a pooled prevalence of IPV during pregnancy in Ethiopia of 26% (confidence interval (CI): 20–32) [6].

### Frameworks of IPV

The multi-level forces which drive IPV in diverse contexts are captured by the integrated ecological framework [7]. Adopted by WHO [8] and later revised to reflect evidence-based risk factors in low and middle-income countries (LMICs) [9], it considers ‘conflict arena’, relationship, male partner, woman, community, and macrosocial factors. Drivers of IPV perpetration [10] were reviewed by the ‘what works to prevent violence against women and girls?’ global programme. Structural factors (poverty, gender inequality, and normalisation of violence in social relationships) interacted with individual and relationship factors (poor communication, poor mental health, substance abuse, child maltreatment, and disability) to drive IPV, exacerbated by armed conflict and post-conflict conditions.

### Perspectives on IPV in Ethiopia

Three qualitative studies have explored IPV in Ethiopia with stakeholders, predominantly community members involved in responding to IPV. In Gondar, in-depth interviews and focus groups (*n* = 46) [11] found that IPV was sometimes considered a sign of love and rationalised by reference to religious teachings. The authors noted a distinction drawn between acceptable conflict and abuse, depending on perceived severity and reasonableness. A second focus group study [12] in East Wollega (*n* = 115) found that IPV was considered a justifiable response to confirmed or suspected infidelity, “failure to give birth”, disobedience, recurrent disagreement, and “controlling and non-domestic” behaviour. All-male arbitration panels encouraged women to withdraw cases, and courts demanded extensive evidence to convict men. A third interview and focus group study (*n* = 30) in Debre Tabor [13] found that the concept of marital rape was often disputed. The study authors attributed the severity of IPV to women’s “failure” to disclose it, indicating a cycle of blame which locates responsibility for IPV within women.

Finally, two studies explored IPV during pregnancy in Jimma. In-depth interviews with 10 male and six female stakeholders [14] found that disadvantages of divorce were cited to justify reconciliation and discourage reporting. Women’s affairs office staff were threatened by abusive partners and obstructed by courts. Ten semi-structured interviews with ANC health workers [15] found that although familiar with physical health and obstetric complications of IPV, clinicians did not discuss mental health impacts or psychological abuse.

### Perinatal mental health in Ethiopia

Several qualitative studies have explored women’s experiences of antenatal and postnatal distress and depression in the Southern Nations, Nationalities and People’s Region (SNNPR) of Ethiopia. In the Butajira health and demographic surveillance site, 25 in-depth interviews and five focus groups were conducted with community stakeholders [16]. IPV was commonly discussed; some women attributed worsening IPV during pregnancy to their increased dependence on their partner. In the same sample [17], participants linked postnatal distress to socio-cultural difficulties. These included pressures in the postpartum period (including expectations to perform specific socio-cultural practices), worsening financial or marital difficulties, and inability to access help due to the practice of postnatal confinement. Inability to earn money rendered postpartum women more dependent on partners, increasing the risk of IPV, resulting in distress and suicidal thoughts.

Qualitative interviews were also conducted in the Sodo district of SNNPR with health workers and pregnant women experiencing depressive symptoms [18]. Women described somatic manifestations of depression during pregnancy, in terms of exhaustion and headaches. Women and health workers conceptualised depression as a state of “thinking too much.” Life difficulties, reproductive health problems, uncompassionate care in labour, and marital conflict were all cited as preoccupations of women who were “thinking too much”.

Although a rich qualitative literature has explored IPV and perinatal mental health in Ethiopia separately, few interview studies have focused on the relationship between women’s experiences of IPV and perinatal emotional difficulties. To inform the adaptation of a brief psychological intervention for rural Ethiopian ANC, we sought to answer the following research questions.

1. What are women’s experiences of IPV during the perinatal period, in this context?
2. What are women and health workers’ perceptions of emotional difficulties during the perinatal period, and their relationship to IPV?

## Methods

### Setting

SNNPR is one of thirteen regions and chartered cities in Ethiopia. The district of Sodo, where this study was conducted, has a projected population of 161,097 [19]. Ethiopian primary care, including ANC, comprises satellite ‘health posts’ staffed by health extension workers (HEWs), health centres staffed by family doctors, nurses, midwives, and health officers, and primary hospitals.

### Study design and methodological orientation

We conducted qualitative in-depth interviews with pregnant women and ANC health workers, to explore their perspectives and experiences of IPV and its relationship to mental health. We adopted the interpretivist view that participants and researchers make subjective interpretations of the social world through their lived experience [20]. We considered our research an inductive process, grounded in the data, albeit influenced by our theoretical orientations.

### Sample

We recruited health workers and pregnant women from health centres and health posts delivering ANC in Sodo and neighbouring Butajira, to triangulate the perspectives of different stakeholders [21]. We recruited a purposive sample to capture the views of women of diverse age, religion, and educational levels, and of health workers of diverse age, religion, specialty, qualifications, and years of experience. The research assistant (RA) asked health workers to identify pregnant women who had reported symptoms of emotional distress, or whom they suspected could be experiencing IPV. Due to the absence of a formal IPV screening system, health workers identified potentially eligible women under their care based on knowledge of their personal circumstances. Potentially-eligible women were also identified from participants in an ANC survey who had reported depressive symptoms on the locally-validated patient health questionnaire [22], and relationship difficulties on a five-item screening questionnaire [23]. We anticipated conducting a total of between 20 and 30 interviews, based on the literature in this field. We determined the point of theoretical saturation through discussion between the first author, last author, and the RA conducting interviews, once the RA noted that new perspectives were not being raised by participants beyond those already captured. The first and last author agreed the point of theoretical saturation, informed by the breadth of perspectives described in existing literature [24].

### Inclusion criteria

Eligible participants were required to be female, aged 16 years or over, pregnant, conversant in Amharic, able to understand the information (discuss the study with the RA and respond to questions about their interest in taking part), and giving informed consent.

We did not formally screen women for IPV exposure, to keep recruitment as inclusive as possible, and avoid excluding women experiencing but not disclosing IPV. Previous qualitative research in the same setting which did not purposively sample women experiencing IPV found that it was frequently described by participants [16–18]. We therefore considered any otherwise eligible pregnant woman to meet inclusion criteria, if her ANC health worker suspected that she was experiencing IPV, or if she had endorsed relationship difficulties on the aforementioned ANC survey. Health workers were required to work in ANC, be conversant in Amharic, and give informed consent.

### Exclusion criteria

Women were excluded from participation if they were acutely unwell, requiring emergency treatment, or the RA noted difficulty in them understanding, remembering, considering the advantages and disadvantages, or communicating their views about the information provided.

### Informed consent

The RA provided eligible women and health workers with written and verbal information (uploaded to an open-access repository: Keynejad [25]) about the study, before inviting them to give written, informed consent. After reading the information sheet or having it read aloud (if unable to read), participants consented by signing or making a thumbprint authorised by a secondary school-educated, independent witness (if unable to write).

### Ethical concerns

The RA ensured that women understood the study by providing written and verbal information in simple language and checking their understanding. The RA was trained to identify verbal and non-verbal cues suggesting that a woman might not feel comfortable with or interested in participating, and to politely cease providing study information to such women. The information sheet explained that women’s responses would be anonymised but excerpts would be shared with others and used to develop an intervention. The voluntary nature of participation was emphasised throughout and could be withdrawn at any time before writing up. This study received ethical approval from King’s College London Psychiatry, Nursing and Midwifery Research Ethics Subcommittee (#HR-17/18–6063), and Addis Ababa University College of Health Sciences (#026/18/Psy).

### Risk management

Interviews followed a standard operating procedure (SOP; see 26) addressing researcher conduct and contact with participants. The SOP provided guidance on how the RA should respond to distress, support participants experiencing IPV, and respond to any risks to children and adults identified during interviews. The SOP guided the RA to take precautions to prevent partners learning about a woman’s participation (for example, reading a leaflet she brought home), maintain confidentiality, seek consent to break confidentiality if required, and urgently discuss concerns with a nominated clinical contact person. The SOP included numbers for local services, to be provided if women felt safe to take written information home.

### Interviewers

Recruitment, consent, and interviewing were conducted by a female, master’s degree-qualified RA, in Amharic. We trained the RA using WHO clinical and policy guidelines on responding to IPV [26]. The RA received regular feedback on early interviews, on responding to verbal cues, and exploring unclear points. The RA kept field notes about interview interactions, which informed analysis.

### Data collection and procedure

In-depth interviews were conducted in a private health centre room. Interviewers collected basic demographic information and followed topic guides, which were employed flexibly [25]. Topic guides addressed relationship problems and violence experienced by pregnant women, and perspectives on asking about mental health and IPV. Interviews also addressed participants’ perspectives on a perinatal mental health intervention, which are reported in a separate study [27]. Participants were reimbursed 100 Ethiopian birr (£2.68), which was considered appropriate locally [28].

### Data handling and protection

All interviews were digitally audio-recorded, downloaded onto a computer, and password-protected, before being emailed to experienced transcribers. Transcribed, password-protected Amharic interviews were then emailed to translators, who emailed de-identified, translated interviews to the RA. Paper documents were stored in a locked cupboard.

### Analysis

We followed the six phase approach to thematic analysis [29]. First author RK familiarised herself with the data by reading and re-reading transcripts alongside field notes, noting initial ideas. We then used NVIVO [30] to systematically code the dataset, generating initial codes and collating relevant quotations. Next, we reviewed initial codes, gathered them into themes, and reviewed themes in relation to constituent quotations. We refined themes, clarifying names and definitions through abstraction and interpretation. In the explanation phase, we referred back to our research questions while writing up.

### Validation and reflexivity

For triangulation through multiple analysis [21], the research team worked closely, for cross-cultural, collaborative ‘sense-making’ [31]. RK selected a transcript from a pregnant woman and one from a health worker, which were particularly rich in content. AM (interviewer), EF (trial coordinator), and TB (post-doctoral researcher) independently familiarised themselves with the data and generated initial codes [29]). RK reviewed these codes, to alter or elaborate initial codes. We remained reflexive of potential differences in the lived experiences of Ethiopian researchers and research participants, which might influence our interpretations. After engaging in initial explanation by sketching out an initial diagram displaying relationships between preliminary themes, RK shared her analysis with AM, EF, TB and CH (a UK researcher living permanently in Ethiopia), before online coding discussions, which informed iterative revisions. RK used reflexivity to identify personal biases, influenced by intersectional personal circumstances [32]. She used self-reflexivity of her position as an English-speaking, female researcher working in a high-income country where gender equality is advocated but not always achieved, to strive for ‘empathic neutrality’ in her interpretations [20].

We did not pursue respondent validation (‘member checking’) of our interpretations. Interviews explored sensitive subjects and there was potential for power imbalances between some participants. Respondent validation could enable more vocal respondents to challenge uncomfortable interpretations, which accurately represented lived realities [21].

We remained mindful that transcripts were English translations of conversations in Amharic. We recalled that meaning conveyed through spoken language incorporates cultural experiences and understandings which cannot always be translated [33].

## Results

### Participant and interview characteristics

Table 1 summarises demographic characteristics of the 16 pregnant women and 12 health worker participants, all of whom were female. Median age was 26.5 years for women and 25 years for health workers. Interviews were conducted between December 2018 and December 2019, and lasted a median 33 minutes (pregnant women) and 35 minutes (health workers).

**Table 1:** Participant characteristics

Characteristic		Pregnant women: n, % (***N*** = 16)	Health workers: n, % (***N*** = 12)
**Age (years)**	16–19	1 (6%)	0
	20–24	4 (25%)	5 (42%)
	25–29	6 (38%)	4 (33%)
	30–34	4 (25%)	3 (25%)
	35–39	1 (6%)	0
**Education**	No formal education	3 (19%)	Not applicable
	Elementary	10 (62%)	Not applicable
	Secondary	3 (19%)	Not applicable
**Religion**	Christian (Orthodox)	10 (63%)	11 (91%)
	Christian (Protestant)	1 (6%)	0
	Muslim	5 (31%)	1 (9%)
**Specialism**	Health extension worker	Not asked	6 (50%)
	Midwife	Not asked	6 (50%)
**Clinical experience (years)**	0–4	Not asked	6 (50%)
	5–9	Not asked	0
	10–14	Not asked	6 (50%)

### Themes

**Fig. 1:**
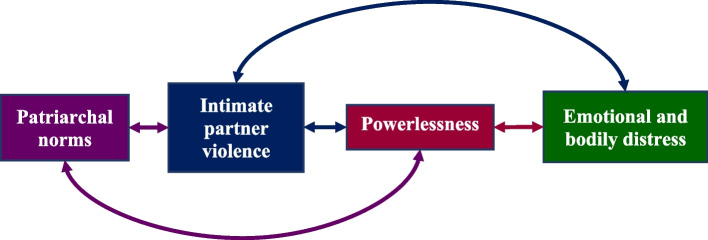
Participant-perceived relationships between themes

Thematic analysis identified abusive experiences, patriarchal norms, powerlessness, and emotional and bodily distress as themes. Figure 1 displays the relationships between each theme, characterised by participants. Arrows reflect participant-perceived associations between themes (boxes). Appendix 1 summarises two auxiliary themes (deprivation and pressures and expectations), which contextualised women’s difficulties but were beyond the scope of this article.

#### Theme 1: “He threatened to cut me into pieces” – intimate partner violence

Participants (P: pregnant women, HW: health workers) described a range of experiences that they considered to be abusive or reinforcing of abuse.

**“He told me that no-one would help me” – abuse.**

IPV was the most extensively elaborated subject across interviews. Experiences included physical, sexual, psychological abuse, and coercive control. Women mentioned the emotional impact of IPV infrequently, usually as a passing comment that “I feel bad” (P7, P9), or “I feel stressed” (P7, P16). Some health workers linked IPV with depression, but minimised its severity. One of the most experienced health workers highlighted inter-relationships between partners’ substance use, poverty, and depression:

*In addition to her being physically abused by her drunk husband, she has financial constraints … household problems and she is responsible to raise the children too, and he doesn’t support her... His abuse added to the economic problem is what caused the depression.*

HW1, HEW, 10–14 years’ experience.

Pregnant women and health workers acknowledged that some women were at risk of femicide but did not discuss the emotional impact of experiencing life-threatening partner violence. One woman described reporting her husband to the police, who suggested that IPV and even femicide were justifiable where infidelity was proven. This was one of several examples of IPV being rationalised. Aggressive interactions with partners’ family members, and actions exacerbating or perpetuating IPV by women’s own relatives were also common. One pregnant woman described how her mother-in-law enabled IPV by giving her husband money for alcohol.

**“He will feel that there is no law” – ineffectual responses** *.*

Participants described a hierarchy of responses to IPV, starting with their own and their partner’s family. When family were unable to help, informal help was sought from neighbours, village elders, or religious leaders. Formal support was sometimes sought from district women and child affairs offices, police, and, finally, the legal system. Women described how authorities enforced incremental pursuit of each step, creating a sense of impunity among perpetrators:

I talked to his family … They took the issue to the [local office] … I went to the court … they told me to go to ‘female and youth affairs’ … she told me that they … first give him advice. It is [later] that they will take him to court … If he gets advice … he will feel there is no law … [it] will not help at all*.*

P2, 30–34 years old.

Police involvement was characterised as a last resort, but only provided women with brief separation without consequences for the perpetrator. Women’s lack of financial independence made prosecution unfeasible, reinforcing the implied futility of seeking help. Both recently qualified and experienced health workers expressed their powerlessness to safely respond to IPV:

In rural areas like this one, women usually have forced sex with their husbands … I think it will be another problem in their marriage if they refuse … It is difficult for me to propose a solution … [Husbands] do that even when they are sober*.*

HW7, Midwife, 0–4 years’ experience.

Options of separation and divorce were mentioned, usually in hypothetical language. Barriers to leaving abusive partners included coercion to stay or return by village elders, mediators, neighbours and friends.

#### Theme 2: “not... the end of the world if a husband beats his wife” – patriarchal norms.

Participants described how perpetration of IPV and other abuse was implicitly reinforced across society.

**“They are dominated” – inequality** *.*

Participants suggested underlying reasons for IPV, such as occupational inequality and gender unequal attitudes, including among women. Health workers described how gender inequality impacted women’s control over decisions about marriage or healthcare (including contraception and termination of pregnancy). Several participants also stated that emasculation or men feeling undermined caused them to be violent:

He doesn’t want anyone to see him when he does the household chores... He advises the [children] or beats them up. He flogs them with his belt*.*

P5, 25–29 years old.

**“Her husband should teach her to behave properly” – victim blaming***.*

The view that women’s behaviour may provoke IPV was common. Some participants said women could prevent IPV by not criticising their partner. Some justified IPV as an appropriate response to women accusing men of infidelity or arguing about money:

It will not be the end of the world if a husband beats his wife … [She] shouldn’t nag him about money … It is easy for women to make plans for fights when they are idle*.*

P10, 25–29 years old.

Participants anticipated community judgement for appearing to violate social norms of unity with one’s husband. Women expressed shame about people knowing they were experiencing IPV, or admitting emotional difficulties and IPV to others. This made women reticent to “divulge secrets” to health workers, further compounding their isolation. Participants emphasised concerns about confidentiality when accessing support, especially in relation to being gossiped about.

**“They are not conscious when they drink” – attribution** *.*

IPV was commonly attributed to situational factors, such as men’s alcohol use, rather than dispositional factors. Men typically drank alcohol after earning money from selling crops at market, provoking arguments about household finances. No participants explicitly linked a man’s decision to drink alcohol with his responsibility for perpetrating IPV. Some participants minimised men’s responsibility for violence while intoxicated; many did not question the imperative to drink socially, or its role in IPV:

He doesn’t want to distance himself from his friends … He acts disruptively only when he comes home drunk … he drinks whether he has money or not*.*

P9, 30–34 years old.

Participants did not raise the possibility that some men may have a violent disposition, or be replicating behaviour witnessed between their own parents during childhood.

**“She should be able to handle the situation wisely” – accommodation** *.*

The perception that it was women’s responsibility to manage their partner’s combativeness was common:

Women could exacerbate the problem when they fail to ignore their husbands … If a wife ignores her husband … he will stop... I think this will help her to avoid [conflict]*.*

HW10, HEW, 10–14 years’ experience.

Women were often advised to leave their partner’s immediate vicinity, for hours, days or weeks. Participants suggested that women should stay with neighbours, move rooms, or leave the home to temporarily avoid their partner. The emotional impact of regularly accommodating IPV was sometimes implied, but not overtly expressed.

**“I usually don’t ask them about that” – looking the other way** *.*

Community members, health services, and authorities routinely ignored, rather than identified and addressed IPV. Some participants minimised the acceptability or likelihood of IPV during pregnancy, due to concerns for unborn children, or women’s vulnerability. Some HEWs and midwives, including several with 10 or more years’ experience, reported never, or infrequently, treating women experiencing relationship problems, alongside idealistic statements about pregnancy and ANC. Several women and some health workers said asking about IPV would be acceptable, but does not happen. Lack of training was the primary reason given.

#### Theme 3: “She cannot do anything to protect herself” - powerlessness

Abusive treatment and its reinforcement by pervasive patriarchal norms left women feeling powerless to change their situation.

**“If I take my children … it will be very difficult for me” – entrapment** *.*

Some women described feeling trapped in abusive relationships because of concern for their children, while others described children mediating between their parents. Short and long-term impacts of IPV on existing children were rarely mentioned. Pregnancy and the welfare of existing children posed barriers to women leaving an abusive relationship. Although some participants suggested that IPV would resolve if women ignored abusive partners, others described it worsening abuse. This form of entrapment highlighted how women received contradictory messages both encouraging assertiveness and discouraging confrontation:

I pretend as if I didn’t see or hear what he did or said … He feels belittled when I don’t confront him when he berates me. He will not stop lashing out … if I don’t confront him*.*

P16, 20–24 years old.

Participants did not describe any emotional impacts of women’s sense of entrapment.

**“There is no way to escape” – hopelessness** *.*

Hopelessness, the inevitability of death, and a lack of meaningful options were commonly expressed. IPV was often said to “have no solution”:

Even her child might be dead due to their conflict … There is no way to escape… Since it is her destiny, she will live with him ‘til the end of her life*.*

P3, 35–39 years old.

Some women expressed the inevitability of their suffering, sometimes in de-humanising terms (“you can do whatever you like with me” (P1)). Occasionally, women described retaliating physically, indicating a hopeless disregard for their safety. Women described how shame, difficulty verbalising and fear of exacerbating IPV made them hopeless about disclosing IPV and emotional difficulties:

I feel ashamed to speak to you because I am not educated like you … I just feel suffocated with fear*.*

P13, 25–29 years old.

#### Theme 4: “I don’t always feel happy … I feel sick” – emotional and bodily distress.

Participants elaborated on perinatal emotional difficulties much less extensively than IPV, and often characterised distress in terms of, or alongside, physical symptoms.

**“I am always anxious” – emotional difficulties** *.*

Some participants raised lack of knowledge about perinatal emotional difficulties as a barrier to disclosing symptoms to health workers. However, women reported anxiety, stress, and depression, which they attributed to exhaustion, relationship conflict, unwanted pregnancies, or to no reason. Symptoms raised included tearfulness, reduced motivation, poor or excessive sleep and nightmares, feeling numb, irritability, poor concentration, and rumination. Two women described suicidal thoughts associated with shame or despair about current or past IPV:

I sometimes want to strangle myself to death … When I think that I am left behind [by my friends] … I have this idea of hurting myself … [Previously] I was hoping to die*.*

P14, 16–19 years old.

**“I go to the health facility every day” – physical symptoms***.*

Participants associated perinatal emotional difficulties with adverse obstetric outcomes, such as intrauterine growth restriction, delivery complications, and stillbirth. Persistent perinatal physical symptoms without identified causes were commonly described. One health worker attributed symptoms to stress and anxiety, managing them with reassurance after ruling out physical pathology. Another (HW3) described a woman with “a tendency to feel dizzy and fall down, losing her consciousness” only when pregnant, which was attributed to “getting stressed out”. Some women were preoccupied with physical symptoms and their perceived mechanisms:

I feel tired and there is a swelling around here, where my heart is … My neighbours … said that the umbilical cord will wrap around the baby’s neck and choke it to death if I sleep like this*.*

P7, 30–34 years old.

Participants did not link persistent perinatal physical symptoms to experiencing IPV.

**“Sadness has an impact on the baby” – responsibility for the unborn** *.*

Women perceived themselves to be responsible for adverse obstetric and neonatal outcomes. Worries about how emotional difficulties could impact their pregnancy included miscarriage, dying in childbirth, coping with a new baby, and impacting the unborn:

A mother’s happiness or sadness has an impact on the baby … The baby is very small … I feel that the baby is suffering from my stress and grief*.*

P14, 16–19 years old.

Participants also described anxiety about physical and obstetric consequences of physical IPV. Adverse obstetric outcomes were attributed to distress caused by IPV, making women still more anxious about the impact of anxiety on their unborn child. Participants alluded to women’s distress in response to IPV impacting foetal well-being:

She could also get very angry. She could get disappointed. Since she is pregnant, her child also will get irritated in her womb*.*

P4, 23 years old.

## Discussion

Our qualitative study in rural Ethiopia found that abusive experiences, emotional and bodily distress were common, and that pregnant women and health workers identified important relationships between them. Contrary to expectations of sensitivity, participants elaborated extensively on IPV. Emotional difficulties were discussed in more restricted terms than IPV, and often conceptualised as part of wider bodily distress. Patriarchal norms explained how community actions in response to IPV often reinforced women’s experiences of abuse. This in turn created a sense of powerlessness to address IPV or change their situation more broadly, exacerbating women’s emotional and bodily distress.

### Perinatal IPV

In our study, IPV was reinforced by norms and experiences at all levels of the integrated ecological framework [7]. Some women described sporadic instances of abuse which they perceived to be isolated events, often associated with their partner being intoxicated with alcohol, interspersed with periods of positive interaction. These women often minimised their partner’s responsibility or the severity of abuse. Intermittent reinforcement within power-imbalanced relationships has been proposed to foster ‘traumatic bonding’ [34]. Traumatic bonding may encourage women to return to abusive partners, because economic and legal barriers to separation are compounded by deprivation of the abusive attachment object.

Childhood factors associated with both women’s victimisation and men’s perpetration of IPV in the revised ecological model [9], such as witnessing parental IPV, were not mentioned. This was despite participants acknowledging that children were exposed to IPV in their own household, and evidence that childhood exposure is a risk factor for IPV perpetration in Ethiopia [35]. Previous research found that corporal punishment of children was legally and culturally acceptable in Ethiopia [36], and that children’s rights, child abuse, and child protection were infrequently reported by Ethiopian media [37]. Future initiatives sensitising communities to IPV should therefore also address impacts of abuse on children. At the female partner level, women linked financial dependence on their partners with IPV, as found by other qualitative studies from sub-Saharan Africa [38, 39].

Links between patriarchal norms, IPV, emotional difficulties, and cyclical community reinforcement of abuse supported the findings of the global ‘what works’ programme [10]. Notably, however, participants did not mention the impact of armed conflict, despite intermittent regional unrest in Ethiopia. Reasons may have included the sense that national and regional armed conflicts were not of direct personal relevance to women’s lives, the chronic nature of episodic unrest in this context or, perhaps, failure to inquire about conflict outside the home in the topic guide. In terms of norms, IPV was often justified through external attribution [40], as an appropriate response to perceived provocation. This is consistent with evidence from other Ethiopian studies, that women are often characterised as provoking abuse (victim blaming) [13, 41]. Studies from Ethiopia and other sub-Saharan African countries also support our findings that IPV was reinforced by a lack of community sanctions [11, 42], unequal gender norms [17, 39, 43], and treating women as property [38, 39].

Community norms meant that women were expected to adjust their behaviour to accommodate IPV, which is consistent with findings from Ethiopia [13] and other sub-Saharan African countries [38, 39, 43, 44]. Interviews highlighted the lack of substantial legal or community repercussions for perpetrators of IPV, as reported by other qualitative studies with women, health workers, and community stakeholders in Ethiopia [12, 14, 15].

In terms of neighbourhood factors, family involvement mirrored abusive intimate relationship dynamics, compounded by ineffectual responses, and coerced reconciliation, as found in other qualitative studies in Ethiopia [12, 14, 15]. At the macrosocial level, participants described pervasive gender inequality, consistent with Ethiopia’s low gender inequality index ranking [45].

Although participants mentioned the existence of divorce they did not perceive it as an option which was realistic or attainable for women, in practice. The difficulty of separation was compounded by community action to return women to abusive partners, as reported by other Ethiopian [11, 14] and sub-Saharan African studies [38, 39, 42]. These findings support evidence that more collectivist societies may respond differently to IPV than individualistic societies, conserving community cohesion at the expense of the individual [9, 46]. Our findings support the need for interventions to address IPV at all levels of the revised ecological framework, especially the often-neglected community and macrosocial factors.

### Perinatal emotional difficulties

In keeping with a study of a different Ethiopian ANC setting [15], we found that the impact of abuse on women’s mental well-being was identified infrequently. Emotional difficulties were often characterised as mild or transient, although their relationship with IPV has been identified by other qualitative studies in this setting [17, 18], other regions of Ethiopia [11], and other sub-Saharan African countries [39, 42, 44]. Health workers focused on the impact of emotional and bodily distress on the pregnancy, in keeping with Ethiopian evidence that ANC health workers considered IPV-related difficulties outside their role [15]. This highlights the need for clinicians in ANC to be trained to ask about and respond, both to emotional difficulties and IPV in this context.

We found that fear of community judgement for perceived failure to comply with social norms exacerbated women’s powerlessness, emotional and bodily distress. This was consistent with qualitative evidence that shame compounds IPV in sub-Saharan Africa [42, 44]. Being gossiped about was a barrier to disclosure, making building trust and confidentiality an important priority for intervention design.

Women and staff often expressed powerlessness to influence IPV or change their broader situation. Amid pervasive reinforcement by patriarchal norms, women’s powerlessness led to feelings of entrapment and hopelessness: potential precursors to depression. This highlighted the importance of building referral pathways between ANC and services which can support women experiencing IPV.

In Ethiopia, shame, humiliation, defeat or entrapment, fatalistic helplessness, and low self-efficacy in relation to an external locus of control were identified in relation to poverty and food insecurity [47]. The authors hypothesised that these processes mediated psychological distress. Deprivation was an auxiliary theme raised spontaneously by participants despite being outside the scope of our study. In very low-income communities, it may not be possible or appropriate to address emotional difficulties or IPV in isolation; a syndemic perspective may be more appropriate [48].

Several participants with personal experience of perinatal emotional difficulties or suicidal ideation associated them with IPV, consistent with Ethiopian [17, 18] and other sub-Saharan African [49] qualitative research. However, symptoms of bodily distress were raised more often than emotions, highlighting the importance of tailoring interventions to women’s explanatory models and idioms of distress .

Community expectations that women should accommodate IPV was linked to self-blame, low self-esteem, and depressive symptoms among some participants, exacerbating their sense of powerlessness still further [34]. Victim blaming, by attributing responsibility for IPV to women’s perceived deficiencies, may also induce internal attribution [40]. Internal attribution to unstable factors (such as ‘provoking’ IPV) is associated with self-blame, tolerating abuse, and reduced likelihood of leaving. Internal attribution to stable factors (such as one’s character) is associated with low self-esteem, hopelessness and helplessness, increasing risks of depression [40]. These findings highlight the need for mental health interventions to be delivered sensitively, to avoid compounding self-blame, and to strengthen women’s self-efficacy.

### Strengths

No previous studies have used qualitative methods to explore the relationship between pregnant women’s mental health and experiences of IPV in Ethiopia in depth. The existing qualitative literature on perinatal mental health in Ethiopia predominantly used focus group discussion methods. Our comparatively large sample of in-depth interviews with a diverse sample of pregnant women and health workers of varied age, religion, and years of experience, overcame the limitations of groups, such as capturing socially desirable responses, neglecting personal perspectives, and divergent viewpoints.

### Limitations

The identification of some potentially eligible participants based on health workers’ knowledge of their circumstances could have over-sampled women experiencing particularly visible or recurrent IPV. However, we also recruited participants disclosing a range of relationship difficulties on an ANC survey. We analysed interview transcripts as English translations, rather than in Amharic. We mitigated for this by attention to our own positionality through reflexivity, and thematic analysis by male and female Ethiopian researchers. Due to the paucity of studies focused on women’s perspectives, we did not interview men or other stakeholders. Exploring the experiences of a sub-sample of participants’ partners could have enhanced interpretation of our findings but could have posed safety risks.

## Conclusions

Although participants were familiar with IPV and perinatal emotional difficulties experienced by women in this rural Ethiopian context, they did not consistently draw links between the two. Our study demonstrated the importance of IPV to emotional difficulties experienced by women in rural Ethiopia during the perinatal period, and the need for mental health interventions to address the ways that IPV impacts women at different levels of the revised ecological framework.

## Supplementary Information

**Additional file 1.** Appendix 1: Auxiliary themes

## Data Availability

Study materials are available, open-access, at an online repository [25]. The datasets generated and analysed during the current study are not publicly available due to the sensitivity of disclosures contained therein but are available from the corresponding author on reasonable request.

## Abbreviations

ANC: antenatal care
HEW: health extension worker
HW: health worker (interviewee)
IPV: intimate partner violence
P: patient (interviewee)
RA: research assistant
RR: relative risk
SNNPR: Southern Nations, Nationalities, and People’s Region (of Ethiopia)
WHO: World Health Organization.
