# Supplementary material for: Pregnant women and health workers’ perspectives on perinatal mental health and intimate partner violence in rural Ethiopia: a qualitative interview study
Source: BMC Pregnancy Childbirth. 2023 Jan 28;23:78. doi: 10.1186/s12884-023-05352-8 (PMC9883891; doi:10.1186/s12884-023-05352-8)
Supplement: Supplementary file 1 — Additional file 1. Appendix 1: Auxiliary themes [file 12884_2023_5352_MOESM1_ESM.docx]

## Appendix 1: Auxiliary themes

Beyond the scope of this article, participants described broader stressors facing pregnant women, in terms of deprivation, pressures and expectations.

### Theme A: “I have no-one to help me” – deprivation

Participants described material, health, and social deprivations, which they characterised as causing and prolonging emotional difficulties, and exacerbating IPV.

#### “Low economic condition” – poverty.

Poverty was prominent, and often linked to stress and anxiety. Participants frequently described hunger, malnutrition, and healthcare costs as stressors. Pregnant women linked financial anxieties to their inability to influence family finances, exacerbated by their husbands’ alcohol use and khat (a stimulant plant) chewing:

When he gets money, he spends it extravagantly. He chews khat… His wastefulness is causing me a mental problem… the bank book doesn’t have my name and picture on it.

P7, 30-34 years old.

Several participants raised women and men’s lack of education as a factor in relationship conflicts. Less educated women were described as lacking awareness of their options and “illiterate” men as not listening to their partners.

#### “There is no-one there where I live” – lack of support.

The second sub-theme of deprivation highlighted women’s lack of practical, economic, and emotional support from their partners, relatives, in-laws, and communities. Some husbands were supportive, and others, both supportive and abusive:

I always feel anxious about life… Though he beats me and treats me unfairly when we have disagreements, he is usually helpful for me… he just berates me when he… becomes disappointed for no reason.

P16, 20-24 years old.

Women also described lacking support from relatives, and their wider community:

The people are not friendly. They will not give food to my kids if I face any problem… the relationship between the people in this area doesn’t go beyond greetings.

P5, 25-29 years old.

Despite wanting support, women expressed social norms of self-sufficiency, reticence to expose their difficulties, and inhibitions about seeking help. Pregnant women and health workers raised isolation from social and emotional support as a source of particular distress. Some women said not having grown up locally, having recently moved, and discouragement by relatives prevented them from building connections. Several women described self-imposed isolation due to guilt, shame, and low mood:

I feel sad and depressed sometimes. I go home and lay down in bed because I worry that I could affect other people’s moods… I feel ashamed… isolate myself from other[s]

P10, 25-29 years old.

Women’s deprivation was compounded by pressures and expectations: the second theme.

### Theme B: “I feared that they would judge me” – pressures and expectations

The second theme highlighted “pressure on women” (P2) to meet high expectations, despite straitened economic circumstances, and other factors beyond their control. Participants described high community standards of propriety, and shame about deviating from norms.

#### “I can’t give them gifts when I go to their houses” – reciprocal obligations.

Despite struggling to cover basic living costs, participants described expectations of reciprocal expenditure around childbirth and Christian orthodox holidays of Meskel and Easter. Women linked inability to fulfil expectations with isolation and relationship conflicts:

His family… don’t like me because I am poor... I don’t visit my family because… I can’t give them gifts when I go to their houses. They don’t visit me because I don’t visit them.

P9, 30-34 years old.

Women described a tension between the collective culture of community support, for example through *idi*r neighbourhood associations, and rejection of those unable to contribute.

#### “He would not be happy with the new child” – unplanned pregnancies.

Avoiding and managing unplanned pregnancies were common expectations of married and unmarried women. Participants linked “giving birth one on top of the other” (HW1) to financial anxiety and difficulty fulfilling domestic roles. Termination was mentioned, but described as difficult to arrange. Health workers described how women felt pressured to avoid closely timed pregnancies, and to conceal contraception. Pregnancy outside marriage caused significant distress, due to conservative values held by Christian and Muslim communities in this setting. Women’s shame was more evident than condemnation of men’s actions.

#### “I don’t want to be idle for more than five days” – fitness to work.

Participants described pressure to work during pregnancy and soon after delivery. Women described difficulty managing chores, working to make and sell *injera* (bread), or traditional alcoholic beverages, throughout pregnancy, even after being advised to stop. Some women and health workers blamed continued manual labour during pregnancy for women missing ANC appointments and for adverse obstetric outcomes.

Women also described pressure to continue working despite headaches, dizziness, back pain, poor sleep, exhaustion, and nausea. Some participants linked high blood pressure with “thinking too much” about the pressures they experienced. Two linked household concerns with anxiety and physical symptoms during pregnancy:

I am feeling very stressed and my nose is bleeding because of that… I feel very anxious… I worry about my household problems and I hold my feelings to myself.

P7, 30-34 years old.
